# Impact of CD4+ T Cell Responses on Clinical Outcome following Oral Administration of Wild-Type Enterotoxigenic *Escherichia coli* in Humans

**DOI:** 10.1371/journal.pntd.0005291

**Published:** 2017-01-19

**Authors:** Monica A. McArthur, Wilbur H. Chen, Laurence Magder, Myron M. Levine, Marcelo B. Sztein

**Affiliations:** 1 Center for Vaccine Development (CVD), University of Maryland, Baltimore, Maryland, United States of America; 2 Department of Epidemiology and Public Health, University of Maryland, Baltimore, Maryland, United States of America; Oxford University Clinical Research Unit, VIETNAM

## Abstract

Enterotoxigenic *Escherichia coli* (ETEC) is a non-invasive enteric pathogen of considerable public health importance, being one of the most common attributable causes of diarrheal illness in infants and young children in developing countries and the most common cause of traveler’s diarrhea. To enhance study-to-study consistency of our experimental challenge model of ETEC in volunteers, and to allow concomitant multi-site trials to evaluate anti-ETEC immunoprophylactic products, hundreds of vials, each containing a standardized inoculum of virulent wild-type (wt) ETEC strain H10407 (serotype O78:H11 expressing colonization factor antigen I and heat-labile and heat-stable enterotoxins), were prepared under current Good Manufacturing Practices (cGMP) and frozen. Following thawing, the contents of each vial can be used (diluted as necessary) to prepare consistent challenge inoculum, even at different study sites. A preliminary human experimental challenge study using this cGMP inoculum was conducted on a research isolation ward and the clinical and cell-mediated immune responses evaluated. Of the 6 healthy adult volunteers challenged 83% (5/6) developed diarrhea and 50% developed moderate-to-severe diarrhea (MSD). Moderate and severe diarrhea were defined as passage of ≥ 1 liter or ≥ 3 liters of diarrheal stool respectively. We compared the CD4+ T cell responses of volunteers who developed MSD against those who did not and identified significant differences in ETEC-specific cytokine production and gut homing potential. We furthermore demonstrated that increased expression of the gut-homing molecule integrin α4β7 by peripheral T follicular helper cells (pT_fh_) correlated with decreased stool volume and increased ETEC-specific IgA B memory cell (B_M_) development. Collectively, despite small numbers of volunteers, our results indicate a potential role for CD4+ T cells, in particular pT_fh_, in modulating disease outcome following exposure to wt ETEC in a volunteer experimental challenge model.

## Author Summary

Enterotoxigenic *Escherichia coli* (ETEC) is an important cause of diarrheal illness in infants and young children in the developing world, as well as in individuals traveling to endemic areas. Due to the lack of suitable animal models for human ETEC infection, we performed a human challenge study in which volunteers ingested wild-type ETEC in a controlled clinical setting. In addition to closely monitoring their clinical status, we studied their ETEC-specific T cell responses prior to and after challenge and studied the presence of associations between CD4+ T cell responses and clinical outcome. We observed differences in the immunological responses of individuals who developed moderate to severe diarrhea following challenge compared to those who did not. These results indicate that T cells may be an important component of the immune response against ETEC.

## Introduction

Enterotoxigenic *Escherichia coli* (ETEC) is one of the most important pathogens contributing to moderate-to-severe diarrhea (MSD) in children in low- and middle-income countries [1]. Moreover, it is also the most common cause of diarrhea among travelers who visit developing countries [2]. In a very recent study, the total estimated ETEC mortality for 2015 was 74,100 deaths in all ages with 23,600 deaths in children < 5 years of age [3]. Following ingestion of infectious ETEC organisms, adhesion to the epithelium is mediated by colonization factor antigens (CFAs), most of which are fimbria or fibrillae [4, 5]. Subsequently, watery diarrhea is induced through the action of heat-stable (ST) and/or heat-labile (LT) enterotoxins. Experimental human challenge studies to investigate the pathogenesis of ETEC diarrhea in humans, study immunity, and evaluate vaccines have been performed at the Center for Vaccine Development (CVD) of the University of Maryland School of Medicine since the mid-1970s, as well as at other institutions [4–16].

ETEC strain H10407, initially isolated in 1971 from the stool of an adult in Bangladesh with severe non-*Vibrio cholerae*, cholera-like, diarrheal illness, produces human ST (STh), porcine ST (STp), and LTh, and expresses colonization factor antigen I (CFA/I) [17]. Although the H10407 strain has been the most commonly used ETEC strain in human challenge studies [18], prior studies were conducted with freshly harvested organisms. Frozen aliquots of challenge inoculum, which can minimize inter- and intra-site variability with attack rates and severity of illnesses, were successfully used with *V*. *cholerae* O1 El Tor Inaba strain N16961 [19]. The availability of these frozen challenge inoculum vials is a precious resource to the scientific community and has been used in efficacy assessments of cholera vaccines [20, 21], including a recently completed pivotal efficacy trial [22].

In preparation for testing the efficacy of immunoprophylactic interventions against ETEC, including both vaccine candidates and monoclonal antibodies, H10407, was fermented under cGMP conditions and identical aliquots were dispensed into several hundred vials that are maintained in GMP frozen storage to provide a source of standardized inocula for future challenge studies. To verify the appropriate challenge inoculum using the frozen material, we conducted a pilot experimental challenge study in volunteers and carefully measured the clinical outcome and immunological responses to experimental ETEC infection.

Despite the global health burden posed by this pathogen, there is currently no licensed vaccine available. Vaccine development has been hindered, in part, by lack of known immunological correlates of protection. Human challenge studies provide an ideal opportunity to investigate the immunological responses which are associated with protection from, or resistance to infection. Due to the non-invasive nature of ETEC, antibodies have been proposed as a major contributor to protection. Thus far, however, few studies have investigated the potential contribution of T cells and their role in protection against this organism. Here we describe, for the first time in humans, CD4+ T cell responses pre- and post-challenge and their association with long-term B memory (B_M_) responses. We further demonstrate an association between T cell reactivity and the susceptibility of participants to develop clinical ETEC diarrheal disease and its severity.

## Methods

### Ethics Statement

The study was approved by the Institutional Review Boards of University of Maryland, Baltimore (UMB), the University of Massachusetts Medical School (UMMS) and the Office of Research Protections of the U.S. Army Medical Research and Materiel Command (since the cGMP production of the challenge inoculum and the clinical trial were supported by the Defense Advanced Research Projects Agency [DARPA] of the U.S. Department of Defense). Written informed consent was obtained from healthy adult volunteers 18 to 49 years of age who were screened for the absence of chronic medical conditions, immunodeficiency, and a history of recent foreign travel to an endemic region or prior cholera or ETEC infection (natural infection or experimental challenge), or prior receipt of a cholera or ETEC vaccine.

### Challenge Strain

A research cell bank (RCB) of ETEC strain H10407 was produced by the Inoculum Preparation Laboratory of the Center for Vaccine Development at the University of Maryland School of Medicine through expansion in animal component-free LB broth (Remel, Lenex, KS). Under cGMP, MassBiologics Manufacturing Facility (Boston, MA) produced the challenge inoculum vials starting from the RCB. Briefly, the RCB was inoculated onto CFA agar plates, incubated at 37°C, and the confluent organisms were harvested. The pooled organisms were aliquoted into gasketed screw-cap cryo-vials (Corning, Tewksbury, MA) and snap frozen; creating a total of 307 vials, each vial containing ~10^10^ viable organisms suspended in phosphate buffered saline (PBS) and 20% glycerol. The frozen challenge inoculum vials are stored at -80°C under a formal stability monitoring plan. This source of cGMP H10407 challenge inoculum has been registered as an investigational new drug (BB-IND-15900) under the U.S. Food and Drug Administration.

### Clinical Challenge

After providing informed consent, six eligible volunteers were admitted as inpatients to a Research Isolation Ward two days prior to challenge to acclimate to the ward and to complete medical screening. On the morning of challenge, a single frozen challenge vial was thawed and diluted to the desired concentration (10^8^ CFU/mL); the inoculum size was confirmed by quantitative counts. Two grams of NaHCO_3_ were dissolved in 150 ml of sterile water and each volunteer (who had been fasting overnight) drank 120 mL of NaHCO_3_ buffer solution; 1 min later each participant ingested ~1 x 10^8^ CFU of the ETEC organisms suspended in the remaining 30 mL of NaHCO_3_ solution.

Following ingestion of ETEC organisms, volunteers were closely monitored for illness and every stool was graded as follows: grade 1, firm; grade 2, soft; grade 3, thick liquid; grade 4, opaque watery; and grade 5, rice water. All stools ≥ grade 3 were considered loose and were weighed. Any individual who developed loose stools was offered oral rehydration salt solution (Jianis Brothers ORS, Kansas City, MO) at a volume of 1.5 times the loose stool volume. If unable to ingest ORS or upon their request, intravenous Lactated Ringers solution was used for rehydration. Ciprofloxacin, 500 mg twice daily for 5 days, was administered when a person exceeded 3 liters of cumulative loose stool output or on day 4 post-challenge, whichever occurred first. Individuals were discharged when they were asymptomatic, completed a course of ciprofloxacin therapy, and demonstrated 3 sequential negative stool cultures for ETEC separated by 12 hours each.

Diarrhea was defined as the passage of 2 or more loose stools (grade 3–5) over a 48 hour period that equaled or exceeded 200 mL or a single loose stool of 300 mL or greater. Moderate and severe diarrhea were defined as the passage of ≥1 liter and ≥3 liters of diarrheal stool, respectively.

Five of the 6 volunteers were Black or African American, 83% were male, and the mean (median) age was 32.7 (32) years.

### Clinical Bacteriology

Stool specimens were plated directly onto Eosin Methylene Blue agar plates (Sigma, St. Louis, MO), MacConkey agar plates (Fisher, Pittsburgh, PA), and GN enrichment broth (Fisher, Pittsburgh, PA), and incubated overnight at 37°C. For each participant a maximum of 2 stools each day were cultured and a rectal swab was obtained if no stool was passed. Colonies suspicious for *E*. *coli* were confirmed by positive agglutination with O78 antisera (Denka Seiken, Campbell, CA) on lactose and indole positive colonies.

### Isolation and *Ex Vivo* Stimulation of Peripheral Blood Mononuclear Cells (PBMC)

Whole blood was obtained from volunteers pre-challenge (day -7), and at multiple time-points after challenge (days 1, 3, 7, 14, 21, 28, 56, and 180 post-challenge). PBMC were isolated from whole blood by Ficoll-Hypaque gradient fractionation and cryopreserved as previously described [23, 24]. All available time-points from an individual volunteer were thawed simultaneously and rested overnight at 37°C 5% CO_2_ in complete media (cRPMI; RPMI 1640 media (Gibco, Carlsbad, CA) supplemented with 100 U/mL penicillin (Sigma), 100 μg/mL streptomycin (Sigma), 50 μg/ mL gentamicin (Gibco), 2 mM L-glutamine (Gibco), 10 mM HEPES buffer (Gibco) and 10% heat-inactivated fetal bovine serum (Gemini Bioproducts, West Sacramento, CA). Of the 6 volunteers challenged with wild-type ETEC, only 5 were evaluable in immunological studies. Following the overnight rest, PBMC were stimulated for 14–18 hours in the absence (media control) or presence of ETEC antigens [whole cell homogenate (5 μl/mL; prepared in-house as described below), LT-B (5 μg/mL; Sigma, St Louis MO), double mutant LT (dmLT) (5 μg/mL; PATH Vaccine Solutions, Seattle WA), and purified CFA/I (5 μg/mL; prepared at the CVD as previously described [25])] or Staphylococcal enterotoxin B (SEB—positive control 10 μg/mL; Sigma) in the presence of Golgi inhibitors (Golgi Plug and Golgi Stop 0.5 μl/mL, BD Biosciences, San Jose CA). Anti-CD107a (LAMP1) monoclonal antibody conjugated to 151Eu (Fluidigm, Sunnyvale, CA) was added to the culture at 3 μl/mL.

Whole cell homogenate was prepared from wt H10407. Briefly, following overnight incubation at 37°C, bacteria were scraped from the plate and suspended in sterile PBS. A microfluidizer was used to homogenize the bacteria followed by heat inactivation at 56°C for 30 minutes.

### Mass Cytometry Measurements

Following overnight stimulation with ETEC antigens or SEB (non-specific positive control), PBMC were stained for mass cytometric analyses using a panel of 23 metal-conjugated monoclonal antibodies. A table of the monoclonal antibodies used is shown in supplementary materials (S1 Table). Briefly, samples were washed once with staining buffer (phosphate buffered saline with 2% FBS and 0.1% sodium azide) and then stained for barcoding at 4°C for 30 minutes using anti-CD45-141Pr (SEB and CFA/I), anti-CD45-154Sm (media and LT-B), or anti-CD45-156Gd (whole cell homogenate and dmLT). Following staining with anti-CD45, sets of 3 samples were combined into a single tube for downstream staining (media/SEB/whole cell homogenate and LT-B/CFA/I/dmLT). Viability staining was performed with cisplatinum (Sigma; 25 μM) for 60 seconds. Following cisplatinum staining, samples were Fc-blocked with human immunoglobulin (Sigma; 3 μg/mL) for 20 minutes at room temperature followed by surface staining for 30 minutes at 4°C. Fixation and permeabilization were performed according to manufacturer’s recommendations (eBiosciences, San Diego CA) followed by intracellular staining for 20 minutes at room temperature. Samples were stained with an Ir191/193 DNA intercalator for cell detection by mass cytometry within 48 hours of sample acquisition and re-suspended in EQ4 normalization beads (Fluidigm). Acquisition was performed using a CyTOF mass cytometer (Fluidigm, formerly DVS Sciences). Data were analyzed with Premium Cytobank. A sample gating strategy for mass cytometric analyses is shown in S1 Fig.

### B Memory (B_M_) Assay

Briefly, frozen PBMC were thawed and expanded with B cell-expansion media consisting of 5 μM 2β-ME (Biorad), 1:100,000 pokeweed mitogen (kindly provided by Dr. S. Crotty), 6 μg/mL CpG-2006 (Qiagen/Operon, Huntsville, AL), 1:10,000 Staphylococcus aureus Cowan (SAC; Sigma–Aldrich, St. Louis, MO) in complete RPMI as previously described [26]. Cells were expanded for 5 days (1×10^6^ cells/well in 6-well plates). The B_M_ ELISpot was performed as follows: Nitrocelulose plates were coated with ETEC antigens [CFA/I (5 μg/mL), LT-B (5 μg/mL), ETEC LPS (5 μg/mL), or dmLT (5 μg/mL)], total goat anti-human IgG (Jackson Immuno Research lab; 5 ug/mL), or total goat anti-human IgA (Jackson Immuno Research lab; 5 ug/mL) diluted in PBS. Plates were incubated overnight at 4°C and blocked with 10% FBS in RPMI-1640 for 2 hours at 37°C. For ETEC antigens, expanded cells were added at 2×10^5^ cells/well in duplicates and incubated for 6 hours at 37°C, 5% CO_2_. For total IgG and IgA, expanded cells were added at 2×10^4^ cells/well in duplicate and serially diluted 2-fold for a total of 7 times. Plates were then washed with PBS-Tween followed by a wash with PBS and incubated with rabbit anti-human biotin conjugated Pan-IgG antibody (Hybridoma Reagent Laboratory, Baltimore, MD) or goat anti-human Pan-IgA antibody (Jackson Immuno Research Labs) overnight at 4°C followed by HRP-conjugated Avidin D (Vector Laboratories, Burlingame, CA) for 1 hour at room temperature. Spots were detected by adding 100 μl/well of 3-Amino-9 etheylcarbazole C (AEC substrate; Calbiochem, La Jolla, CA) at room temperature in the dark. The reaction was stopped after 8–10 minutes by washing with distilled water. Spots were counted in an automated ELISpot reader (Immunospot, Cellular Technology Ltd., Cleveland, OH) and analyzed using the Immunospot version 5.0 software. Results are expressed as percent of antigen-specific spot forming cells (SFC) per total IgA or IgG SFC.

### Statistical Analyses

Comparisons between Resistant and Susceptible volunteer groups were performed using unpaired t test in GraphPad Prism version 6.0 (Graphpad Software, San Diego, CA). Associations between integrin α4β7 expression and stool volume or ETEC-specific IgA B_M_ were determined using linear regression in GraphPad Prism version 6.0. For analyses that involved repeated observations from the same volunteer over time, we used random effects models (as fit by SAS Proc Mixed) to account for the correlation between repeated measures from the same person.

## Results

### Clinical Response to Challenge

Within 18 hours from ingesting the challenge inoculum, volunteers began to produce loose stools. There was a 50% (3 of 6) attack rate for MSD (Table 1). Two volunteers achieved the definition of severe diarrhea (≥3 liter) and received early antibiotic intervention. One volunteer achieved moderate diarrhea (≥1 liter) and two volunteers had mild diarrhea and both achieved this on the fourth day post-challenge. Upon initiation of antibiotics, symptoms and shedding of culturable organisms ceased rapidly, within 1–2 days. One volunteer (number 4016) did not experience any diarrhea, but culturable ETEC organisms were detected in his/her stool specimens on the first day post-infection. Additional signs and symptoms associated with challenge are described in S2 Table. For the purpose of examining the cell-mediated immune responses in these participants, we assigned the designation of “Susceptible” to those who experienced MSD. Those who experienced mild (<1 liter) or no diarrhea were designated “Resistant”.

**Table 1:** Individual diarrheal purge to challenge.

	Daily diarrhea volume and shedding of ETEC post-challenge									
Vol #	Day 1	Day 2	Day 3	Day 4	Day 5	Day 6	Day 7	Cumulative Diarrheal Volume (mL)	Cumulative No. of Diarrheal Stools	Clinical Outcome
**4016**	0	0	0	0	0	0	0	0	0	Resistant
	**+**	-	-	-	-	-	-			
**4006**	0	49	99	269	91	89	0	597	9	Resistant
	**+**	**+**	+	+	-	-	-			
**4001**	0	146	109	186	219	0	0	660	6	Resistant
	**+**	**+**	-	-	-	-	-			
**4015**	0	0	913	393	60	0	0	1366	11	Susceptible
	-	**+**	**+**	**+**	-	-	-			
**4008**	1102	1141	1106	323	65	0	0	3737	15	Susceptible
	**+**	**+**	**+**	**+**	-	-	-			
**4009**	567	2043	1066	159	88	0	0	3923	20	Susceptible
	**+**	**+**	**+**	**+**	-	-	-			

### CD4+ T Cell Responses following ETEC Challenge

**Fig 1:**
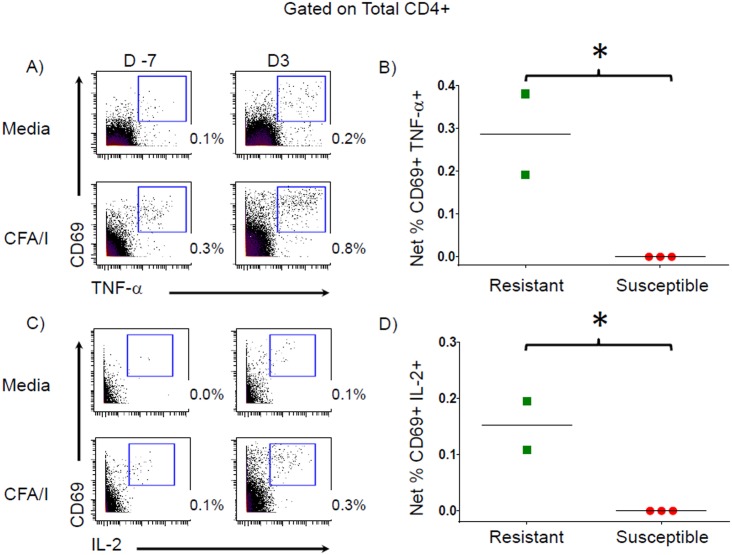
Cytokine production by total CD4+ T cells. **A & C)** Representative histograms, from a Resistant volunteer, demonstrating TNF-α **(A)** and IL-2 **(C)** production by total CD4+ T cells following stimulation with CFA/I in the presence of Golgi inhibitors. **B & D)** Net TNF-α **(B)** and IL-2 **(D)** production (CFA/I stimulated minus media), D3 –pre-vaccination, by total CD4+ T cells in Resistant (green squares) and Susceptible (red circles) volunteers. * p < 0.05

PBMC from challenged volunteers were stimulated with ETEC antigens followed by mass cytometric analyses to investigate the CMI responses. There were no differences in the % of total CD4+ T cells, T effector memory (T_EM_), T_EM_ CD45RA+ (T_EMRA_), T central memory (T_CM_) or T naïve (T_N_) populations between volunteers who developed MSD (Susceptible) and those who did not (Resistant) (S2 Fig). To identify T cells responding to ETEC antigens, we measured cytokine production by CD4+ T cells stimulated with CFA/I, whole cell homogenate, LT-B, and dmLT. PBMC from Resistant volunteers produced significantly increased levels (above baseline) of net TNF-α (0.2–0.4%; p = 0.027 unpaired t test) and IL-2 (0.1–0.2%; p = 0.018 unpaired t test) on day 3 post infection in response to *in vitro* stimulation with CFA/I while Susceptible volunteers did not show increased cytokine production above baseline when stimulated with CFA/I (Fig 1). Responses to the other ETEC antigens did not differ significantly between Resistant and Susceptible volunteers with the exception of low levels of whole cell homogenate-specific IL-2 production (0.05%) which was significantly higher in Resistant than Susceptible volunteers (p = 0.041, unpaired t test) (S3 Fig).

Since the strongest responses were noted against CFA/I, we measured the expression of CD154 (CD40L), as well as IFN-γ and IL-17A production following CFA/I stimulation (S4 Fig). While volunteers who were Resistant showed trends towards increases above pre-challenge in net CD154 expression and IFN-γ and IL-17A production on day 3 post-challenge, Susceptible volunteers did not (S4A, S4B and S4C Fig).

**Fig 2:**
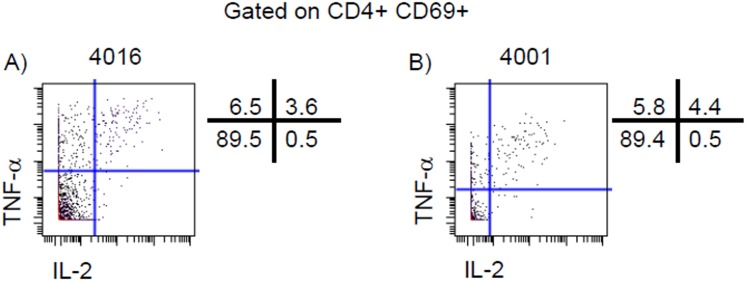
Multifunctional CD4+ T cells. TNF-α and IL-2 multifunctional CD4+ T cells following CFA/I stimulation on day 3 post infection in Resistant volunteers **A)** 4016 and **B)** 4001.

We further explored the multifunctionality of CFA/I-specific CD4+ T cell responses in the 2 Resistant volunteers, and identified TNF-α single positive cells as well as TNF-α and IL-2 double positive cells (Fig 2). To determine the CD4+ T cell subsets responsible for CFA/I-specific cytokine production, we calculated the percentage of cytokine response attributable to the 4 memory subsets (T_EM_, T_EMRA_, T_CM_, and T_N_) as well as to pT_fh_ (Table 2). As expected, the majority of the cytokine production (57–85%) was attributable to the T_EM_ subset.

**Table 2:** Percentage of Total CD4+ CFA/I-specific effector responses on day 3 post-challenge attributable to T_M_ and pT_fh_ subsets in Resistant volunteers. Total pT_fh_ represented 17.0% and 8.6% of total CD4+ T cells in volunteers 4016 and 4001, respectively.

Cytokine	Volunteer	α4β7+	α4β7−
IL-2	4016	33	67
	4001	27	73
TNF-α	4016	32	68
	4001	21	79

**Fig 3:**
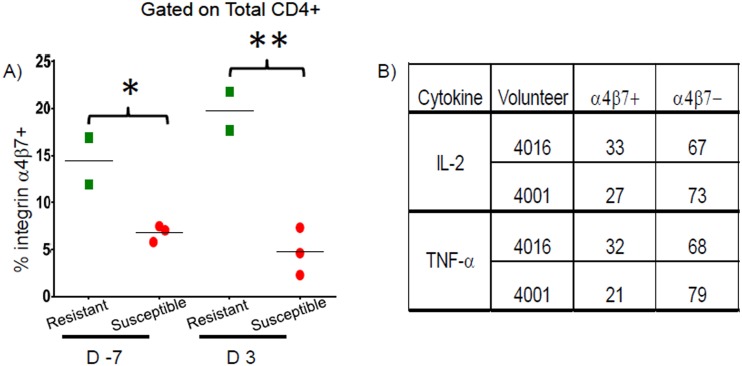
Percentages of total CD4+ expressing integrin α4β7 in Resistant and Susceptible volunteers and percentages of cytokine responses attributable to integrin α4β7+ and integrin α4β7- populations in Resistant volunteers. **A)** Percentages of total CD4+ T cells expressing integrin α4β7 following stimulation with CFA/I at baseline (D -7) & 3 days post-challenge (D3) are shown. **B)** Percentages of total CD4+ CFA/I-specific effector responses on day 3 post-challenge attributable to integrin α4β7+ and integrin α4β7- subsets in Resistant volunteers. * p < 0.05 ** p < 0.01

As ETEC is an enteric pathogen, we also investigated the expression of the gut-homing molecule integrin α4β7 by CD4+ T cells. We identified significantly higher percentages of CD4+ T cells expressing integrin α4β7 prior to challenge and at 3 days post-challenge (p = 0.030 and p = 0.009 respectively, unpaired t test) in Resistant volunteers (Fig 3A). In Resistant volunteers, TNF-α and IL-2 were produced by CD4+ T cells with and without gut-homing potential (Fig 3B).

### pT_fh_ Responses following ETEC Challenge

**Fig 4:**
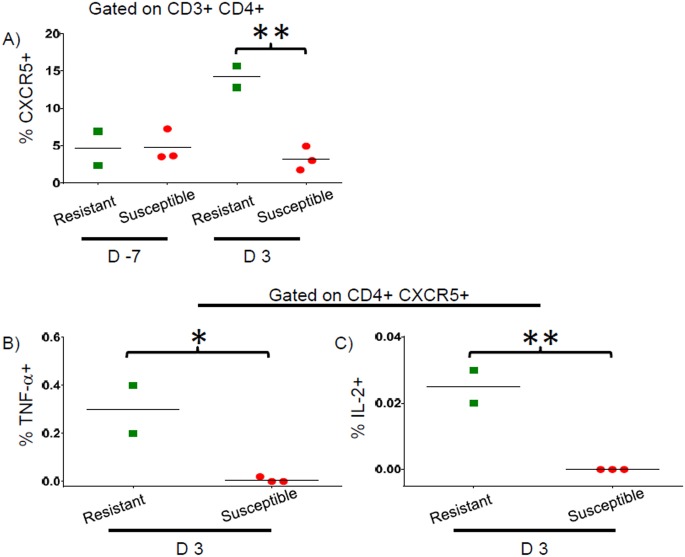
Percentage of pT_fh_ and cytokine production by pT_fh_ in Resistant and Susceptible volunteers. **A)** Live single cell CD14- CD19- events were gated on CD3, followed by gating for CD4 and then CXCR5. The percentage of CD4 cells positive for CXCR5 is indicated at baseline (D -7) and 3 days post challenge (D 3). **B)** Net CFA/I-specific TNF-α production above pre-challenge (D -7) by CD4+ CXCR5+ (pT_fh_) on day 3 post-challenge (D3). **C)** Net CFA/I-specific IL-2 production above pre-challenge (D -7) levels by CD4+ CXCR5+ (pT_fh_) on day 3 post-challenge (D3). * p < 0.05 ** p < 0.01

Because of the great importance of pT_fh_ in contributing to antigen-specific antibody responses [27], we then measured the percentages of pT_fh_ in Resistant and Susceptible volunteers prior to and at 3 days post-challenge. Of note, we identified significantly higher percentages of CXCR5+ pT_fh_ in post-challenge specimens of Resistant volunteers compared to Susceptible volunteers (p = 0.006, unpaired t test) (Fig 4A). There were also detectable increases in CFA/I-specific TNF-α and IL-2 production by pT_fh_ on day 3 post-challenge (above baseline) in Resistant volunteers but not Susceptible volunteers (Fig 4B & 4C). Furthermore, 12–17% of the CFA/I-specific cytokine production by CD4+ T cells was attributable to pT_fh_ which represented 8.6–17% of the total CD4+ population (Table 2). As seen in total CD4+ T cells, there were no significant differences in the increases observed above pre-challenge levels for net production of IFN-γ, IL-17A, or IL-21 or for CD154 or ICOS expression at day 3 post-challenge (S5 Fig).

**Fig 5:**
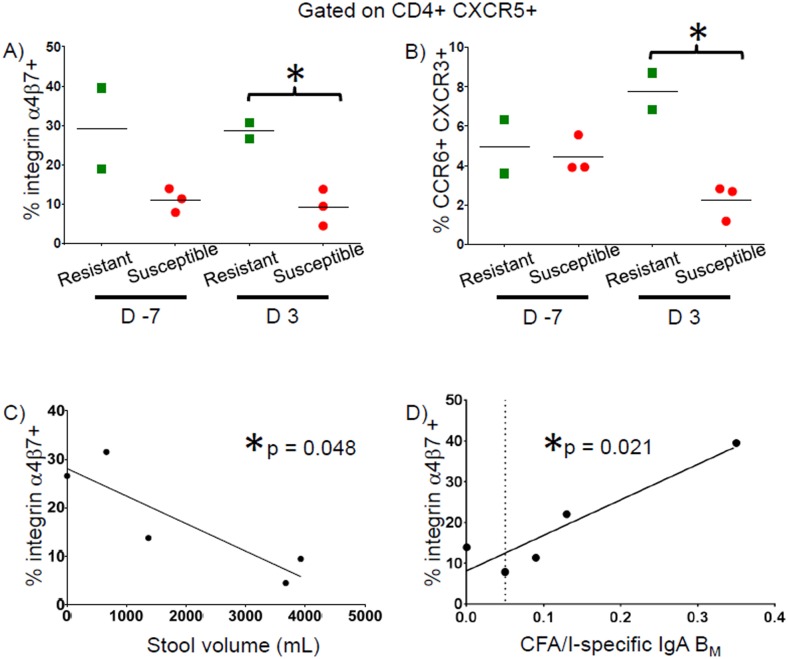
Percentages of pT_fh_ expressing integrin α4β7 and other homing molecules in Resistant and Susceptible volunteers and association with stool volume and IgA B_M_. **A)** Percentages of pT_fh_ (CXCR5+) expressing integrin α4β7 (integrin α4β7+ CCR4-) following stimulation with CFA/I at baseline (D -7) and 3 days post-challenge (D3). **B)** Percentages of pT_fh_ (CXCR5+) expressing CCR6 and CXCR3 (CCR6+ CXCR3+) following stimulation with CFA/I at baseline (D -7) & 3 days post-challenge (D3). **C)** Linear regression analysis comparing the percentages of pT_fh_ expressing integrin α4β7 (integrin α4β7+ CCR4-) following CFA/I stimulation on day 3 post-challenge versus cumulative stool volumes. **D)** Linear regression analysis comparing the percentages of pT_fh_ expressing integrin α4β7 (integrin α4β7+ CCR4-) following CFA/I stimulation pre-challenge (D -7) versus CFA/I-specific IgA B_M_ as a percent of total IgA B_M_ on day 21 post-challenge. * p < 0.05

We further explored the homing potential of pT_fh_ and identified significantly higher percentages of pT_fh_ expressing integrin α4β7 or co-expressing CCR6 and CXCR3 (p = 0.014 and p = 0.011 respectively, unpaired t test) in Resistant volunteers on day 3 post challenge (Fig 5A & 5B). Moreover, expression of integrin α4β7 by pT_fh_ was inversely correlated with cumulative stool volume (Fig 5C; p = 0.048, linear regression). We also investigated the association of integrin α4β7 expressing pT_fh_ at early time-points with later IgA B_M_ responses. Volunteers with higher percentages of integrin α4β7 expressing pT_fh_ prior to challenge had more robust CFA/I-specific IgA B_M_ responses on day 21 post challenge (Fig 5D; p = 0.021, linear regression).

### ETEC-Specific B_M_ Responses

**Fig 6:**
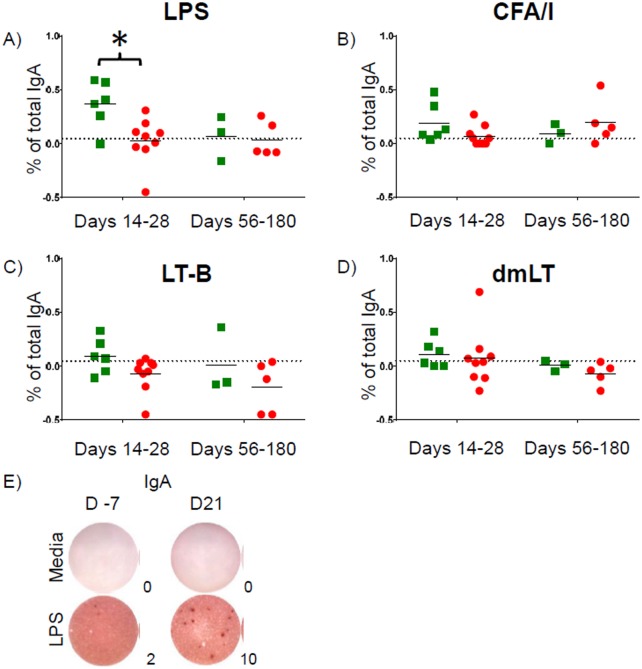
ETEC-specific B_M_ responses. Net (minus baseline) IgA B_M_ responses against **A)** LPS, **B)** CFA/I, **C)** LT-B, and **D)** dmLT expressed as percentage of total IgA B_M_ responses. Points above the dashed line (0.05% of total) indicate responders. **E)** Representative example of B_M_ ELISpots. * p < 0.05

All evaluable volunteers mounted IgA B_M_ responses against at least one ETEC antigen. We identified higher ETEC LPS-specific B_M_ responses (as the increase in % of total IgA over baseline) in Resistant volunteers during days 14–28 post-challenge; however, these differences were not evident at later time-points (days 56–180 post-challenge) (Fig 6A & 6E). We did not detect differences in B_M_ responses between Resistant and Susceptible volunteers against any of the other antigens tested (CFA/I, LT-B, and dmLT) (Fig 6B–6D)

We also found that higher integrin α4β7 expression by pT_fh_ following stimulation with whole cell homogenate correlated with net IgA B_M_ responses to LPS antigen on day 14 post-challenge (p = 0.025, linear regression) and approached significance on day 28 post-challenge (p = 0.08, linear regression) (S6 Fig).

## Discussion

A DARPA grant supported the manufacture of a cGMP standardized inoculum of ETEC strain H10407 to assess the efficacy of candidate anti-ETEC immunoprophylactic products, thereby enhancing study-to-study consistency at the CVD and offering the possibility of concomitant testing at multiple clinical challenge sites. DARPA also supported a small clinical trial to document that the frozen cGMP inoculum was capable of causing typical ETEC diarrhea. This challenge study, in turn, provided an opportunity to measure the humoral and cell-mediated immune responses following challenge. Specifically, to look for reactivity of CD4+ T cells collected pre- and post-challenge, to determine their response following *in vitro* exposure to ETEC antigens and whether T cell reactivity was associated with some degree of resistance to developing ETEC diarrhea or to manifesting milder illness. Volunteers were recruited from the Baltimore-Washington area. A history of recent foreign travel to an endemic region, prior cholera or ETEC infection (natural infection or experimental challenge), or prior receipt of a cholera or ETEC vaccine were exclusionary criteria. Although the challenged volunteers represent an ETEC-naïve population in terms of travel, U.S. residents are likely repetitively exposed to low doses of either animal or human ETEC through fruits and salad vegetables imported from Mexico and Central American countries where ETEC is endemic, and perhaps from U.S. beef and pork products having low level contamination with animal ETEC [28, 29]. Thus, non-travelled members of the U.S. population may have had prior exposure to ETEC antigens. At a challenge dose of ~1 x 10^8^ CFU, half of the volunteers challenged with ETEC developed MSD. This dichotomy provided an opportunity to explore differences in immune responses between volunteers who were Resistant and those who were Susceptible.

Due to the non-invasive nature of ETEC, it is hypothesized that humoral responses play a major role in protection and much evidence supports this contention. Volunteers pre-treated with immunoglobulin from the milk of cows hyper-immunized with an array of ETEC pathogens that included different serotypes expressing the major CFAs and LT were 100% protected compared to a diarrhea attack rate of 90% in controls when challenged with ETEC strain H10407. Based on these and similar observations, an antibody preparation from the milk of cows immunized with ETEC is a prophylactic product against ETEC for travelers with evidence of efficacy coming from three separate volunteer challenge studies [30]. In contrast, very limited information is available on the contributions of T cells, which are likely to play a major role in promoting antibody responses. CD4+ T cells perform multiple critical functions within the immune system, including provision of B cell “help”, enhancement and maintenance of CD8+ T cell responses, and regulation/suppression of immune responses to maintain homeostasis [31]. Production of cytokines, i.e., TNF-α, IL-2, IFN-γ, and IL-17A, in response to antigenic stimulation is an important function of CD4+ T cells. Although there were no significant differences in the percentages of total CD4+ T cells or CD4+ T cell memory subsets between Resistant and Susceptible volunteers, we identified significantly higher increases above baseline in CFA/I-specific production of TNF-α and IL-2 at day 3 post-challenge by CD4+ T cells in volunteers who did not develop MSD (Resistant) following challenge. Furthermore, these responses were multifunctional, a potential indicator of higher quality responses [32, 33]. TNF-α is a potent pro-inflammatory cytokine and plays an important role in recruiting polymorphonuclear and other immune cells to sites of inflammation, while IL-2 plays a critical role in influencing the proliferation, differentiation, and survival of antigen-specific T cells [34]. Together, these cytokines may play a significant role in a protective immune response against ETEC. Although IFN-γ secretion has been documented following ETEC vaccination [35], we did not identify a significant difference in IFN-γ production between Resistant and Susceptible volunteers. It is important to note that measurement of IFN-γ secretion by Wenneras *et al* was by ELISPOT and ELISA which cannot distinguish among the specific cell subset(s) producing the cytokine. It is therefore possible that other cell types, besides CD4+ T cells, are responsible for producing IFN-γ in response to CFA/I stimulation. In fact, IFN-γ has been shown to be produced by natural killer cells (NK), macrophages, dendritic cells, and B cells [36].

Cytokine production in response to other ETEC antigens (whole cell homogenate, LT-B, and dmLT) was not as pronounced as the responses observed following CFA/I stimulation. It is important to note that the challenge inoculum was grown on CFA agar which may have favored the expression of CFA/I. PBMC from Resistant volunteers demonstrated increases above baseline in production of both TNF-α and IL-2 by CD4+ T cells following stimulation with whole cell homogenate which contains a broad array of ETEC antigens (likely including CFA/I, LPS, LT and ST, pili, etc). The role of CFAs in ETEC pathogenesis has been well documented [37] and CFA/I is a major fimbrial antigen which has been shown to induce IgA antibodies following infection [38]. In the present study, we found CFA/I to be the strongest stimulator of cytokine responses in PBMC from Resistant individuals, indicating that this may be an important antigen for inclusion in future vaccine development.

Integrin α4β7 is an important molecule associated with homing of lymphocytes to the gut, the site of initial encounter with ETEC [39]. Importantly, we identified significantly higher levels of integrin α4β7 expression on CD4+ T cells in volunteers who were Resistant, indicating that the ability of CD4+ T cells to home to the sight of pathogen encounter may play a role in protection. We further identified CFA/I-specific cytokine producing CD4+ T cells within both the integrin α4β7 positive and negative populations, suggesting that exposure to wt ETEC elicits specific CD4+ cells capable of homing to the gut as well as to extra-intestinal sites.

pT_fh_ are a circulating subset of CD4+ T cells characterized by the expression of CXCR5 and responsible for promoting antibody production by B cells [40]. In fact, the expression of different homing molecules by pT_fh_ has been reported to affect the class of antibodies produced by B cells (i.e., CCR6 expressing pT_fh_ cells have been shown to play a role in IgA production) [40]. Here we describe, for the first time, the expression of integrin α4β7 by pT_fh_ indicating that this subset has the potential to home to the gut. Furthermore, expression levels of integrin α4β7 on pT_fh_ were higher in Resistant volunteers than Susceptible volunteers at day 3 post challenge and higher levels of integrin α4β7 expression by pT_fh_ were associated with lower cumulative stool volumes. Moreover, early expression of integrin α4β7 by pT_fh_ correlated with ETEC-specific IgA B_M_ responses. Interestingly, CFA/I-specific integrin α4β7 expression by pT_fh_ correlated with increased CFA/I-specific IgA B_M_ responses, while whole cell homogenate-specific integrin α4β7 expression by pT_fh_ correlated with increased LPS-specific IgA B_M_ responses. These results support the concept that interaction between antigen-specific pT_fh_ and their B cell counterparts in the gut results in antigen-specific B_M_ responses which may confer long-term protection in humans.

While this is the first report of integrin α4β7 expression by pT_fh_, we have previously reported that oral immunization of volunteers with attenuated oral *S*. Typhi vaccines or challenge with wild-type *S*. Typhi, another human-restricted Gram negative enteric bacterial pathogen, elicits *S*. Typhi-specific T_EM_ and T_reg_ which expressed, or not, the gut homing molecule integrin α4β7 [41–44]. Additionally the gut homing potential of *S*. Typhi-specific T regulatory cells is associated with increased susceptibility to typhoid disease in a challenge model [42] highlighting the importance of pathogen-specific cells homing to the site of initial antigen encounter.

It is, at present, unknown why volunteers with no known previous ETEC exposure (through natural infection or immunization) would be Resistant to infection in this challenge model. It is possible that Resistant volunteers have some pre-existing immunity secondary to subclinical exposure to ETEC in the past, or, alternatively, exposure to cross-reactive antigens from related Gram negative organisms such as those in the gut microbiota. It is also possible that volunteers with higher levels of integrin α4β7 expression on circulating CD4+ T cells have a more robust T cell response at the mucosal level resulting in lack of moderate to severe disease. The mechanisms by which volunteers are protected from ETEC challenge may be further elucidated by studies in which volunteers are vaccinated against and then challenged with wt ETEC.

An important limitation of the present study is the small sample size. However, we identified several important statistically significant differences between groups of volunteers who were Resistant and Susceptible. Upcoming clinical trials involving challenges with wt ETEC in larger numbers of volunteers will be directed to confirm and extend the novel findings presented herein. Additionally, challenges preceded by immunization will allow us to better define the protective immune responses induced by ETEC vaccine candidate(s).

## Supporting Information

S1 Table Monoclonal antibodies used. The monoclonal antibodies (including clones) and the metals to which they were conjugated are listed.
(TIF)

S2 Table Summary of clinical reponses to challenge. The number and percent of volunteers with each clinical feature are listed.
(TIF)

S1 Fig Gating Strategy for mass cytometry data. Normalization beads were excluded, followed by selection of single cells based on DNA content. Viability was determined using cisplatinum (195Pt). Monocytes and B cells were excluded (CD14 and CD19 respectively). Cells were then gated on CD3, followed by gating on CD4 and CD8.
(TIF)

S2 Fig Percentage of total CD4+ T cells and memory subsets in Resistant and Susceptible volunteers. **A)** Live single cell CD14- CD19- events were gated on CD3, followed by gating for CD4. The percentage of CD3 cells positive for CD4 are indicated at baseline (D -7) and 3 days post challenge (D 3). **B-E)** CD3+ CD4+ T cell memory subsets at baseline (D -7) and 3 days post challenge (D 3). **B)** T_EM_: CD45RA- CD62L-, **C)** T_EMRA_: CD45RA+ CD62L-, **D)** T_CM_: CD45RA- CD62L+, **E)** T_N_: CD45RA+ CD62L+
(TIF)

S3 Fig Cytokine production by total CD4+ T cells. **A & B)** Net TNF-α **(A)** and IL-2 **(B)** production (ETEC antigen stimulated minus media), D3 –pre-vaccination, by total CD4+ T cells in Resistant (green squares) and Susceptible (red circles) volunteers. * p < 0.05
(TIF)

S4 Fig Activation of and cytokine production by total CD4+ T cells. **A-C)** Net CD154 expression **(A)**, IFN-γ **(B)**, and IL-17A **(C)** production (CFA/I stimulated minus media), D3 –pre-vaccination, by total CD4+ T cells in Resistant (green squares) and Susceptible (red circles) volunteers.
(TIF)

S5 Fig Activation of and cytokine production by pT_fh_. **A)** Net CD154 expression **B-D)** Net production of IFN-γ **(B**), IL-17A **(C)**, IL-21 **(D)**, and net ICOS expression **(E)** (CFA/I stimulated minus media), D3 –pre-vaccination, by pT_fh_ in Resistant (green squares) and Susceptible (red circles) volunteers.
(TIF)

S6 Fig Association of integrin α4β7 expression by pT_fh_ and IgA B_M_. Linear regression analysis comparing percentages of pT_fh_ expressing integrin α4β7 (α4β7+ CCR4-) following stimulation with whole cell homogenate on day 3 post-challenge versus the LPS-specific IgA B_M_ as a percent of total IgA B_M_ on **(A)** day 14 and **(B)** day 28 post-challenge.
(TIF)

S1 Checklist STROBE Checklist. (PDF)
