# Supplementary figures and images for: Impact of CD4+ T Cell Responses on Clinical Outcome following Oral Administration of Wild-Type Enterotoxigenic Escherichia coli in Humans
Source: PLoS Negl Trop Dis. 2017 Jan 19;11(1):e0005291. doi: 10.1371/journal.pntd.0005291 (PMC5283752; doi:10.1371/journal.pntd.0005291)

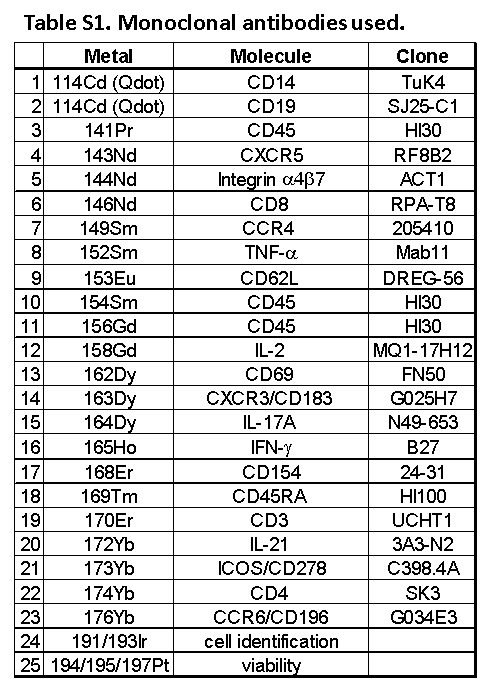

Supplement: S1 Table — The monoclonal antibodies (including clones) and the metals to which they were conjugated are listed. (TIF) [file pntd.0005291.s001.tif]

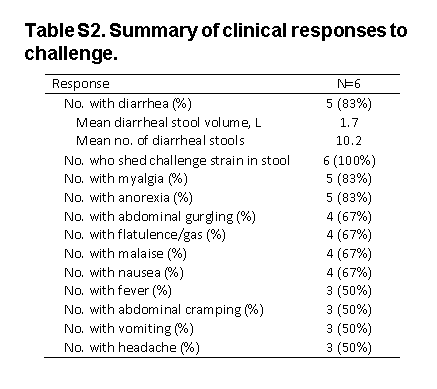

Supplement: S2 Table — The number and percent of volunteers with each clinical feature are listed. (TIF) [file pntd.0005291.s002.tif]

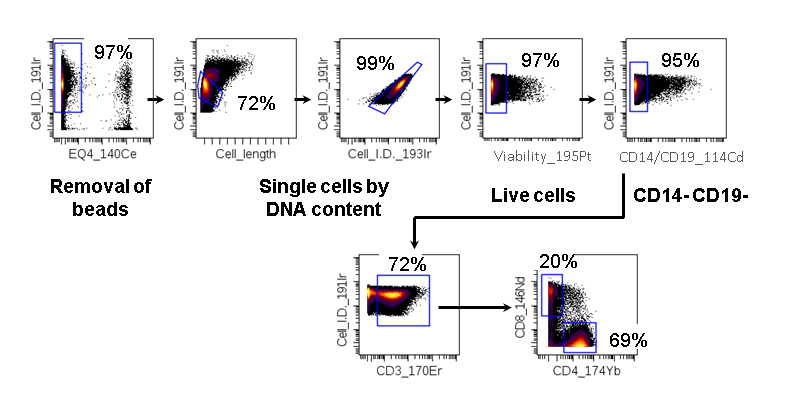

Supplement: S1 Fig — Normalization beads were excluded, followed by selection of single cells based on DNA content. Viability was determined using cisplatinum (195Pt). Monocytes and B cells were excluded (CD14 and CD19 respectively). Cells were then gated on CD3, followed by gating on CD4 and CD8. (TIF) [file pntd.0005291.s003.tif]

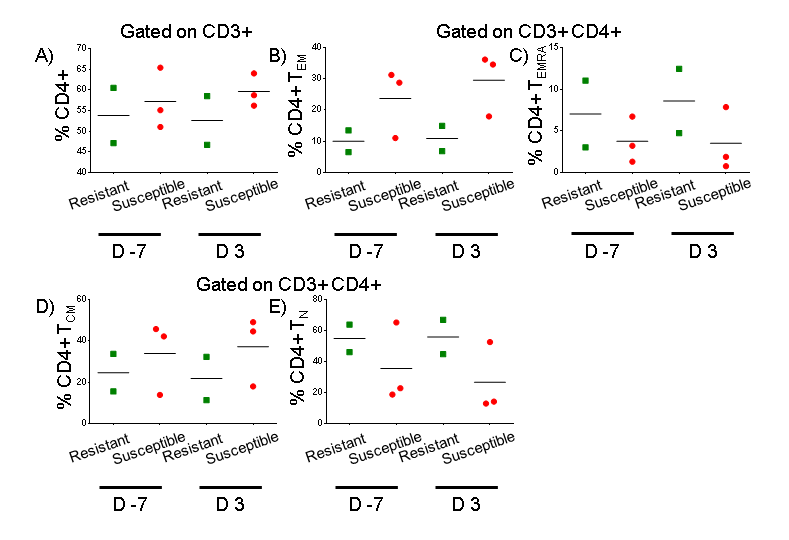

Supplement: S2 Fig — A) Live single cell CD14- CD19- events were gated on CD3, followed by gating for CD4. The percentage of CD3 cells positive for CD4 are indicated at baseline (D -7) and 3 days post challenge (D 3). B-E) CD3+ CD4+ T cell memory subsets at baseline (D -7) and 3 days post challenge (D 3). B) TEM: CD45RA- CD62L-, C) TEMRA: CD45RA+ CD62L-, D) TCM: CD45RA- CD62L+, E) TN: CD45RA+ CD62L+ (TIF) [file pntd.0005291.s004.tif]

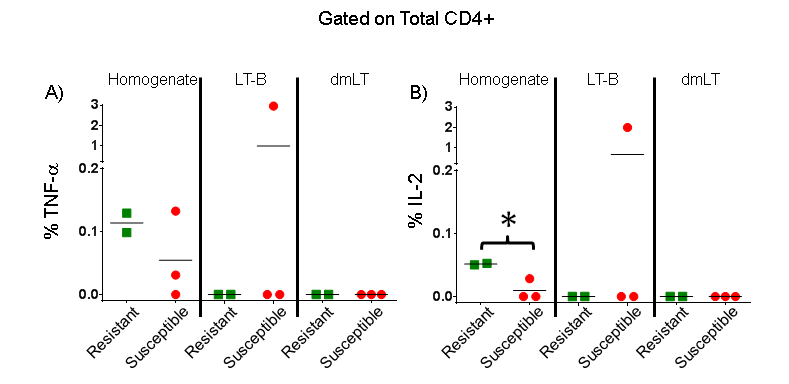

Supplement: S3 Fig — A & B) Net TNF-α (A) and IL-2 (B) production (ETEC antigen stimulated minus media), D3 –pre-vaccination, by total CD4+ T cells in Resistant (green squares) and Susceptible (red circles) volunteers. * p < 0.05 (TIF) [file pntd.0005291.s005.tif]

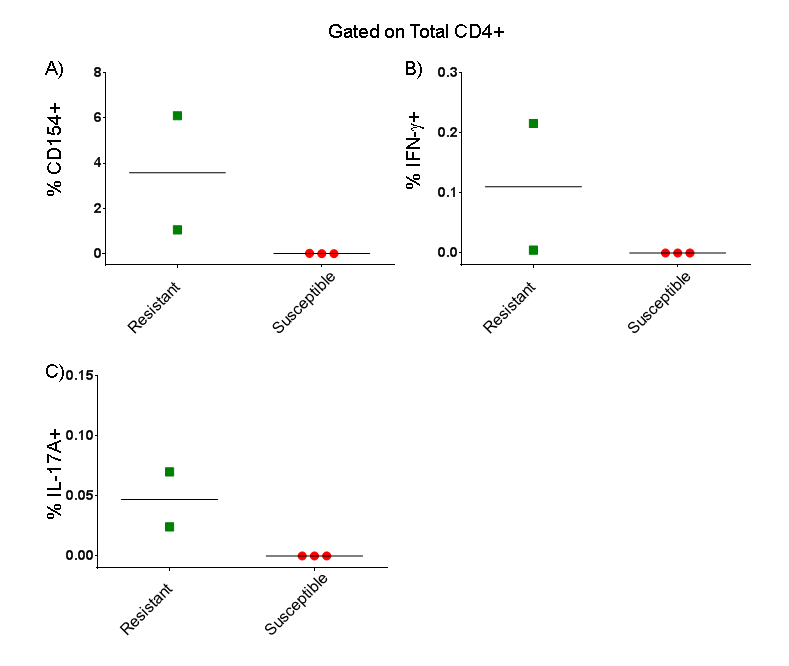

Supplement: S4 Fig — A-C) Net CD154 expression (A), IFN-γ (B), and IL-17A (C) production (CFA/I stimulated minus media), D3 –pre-vaccination, by total CD4+ T cells in Resistant (green squares) and Susceptible (red circles) volunteers. (TIF) [file pntd.0005291.s006.tif]

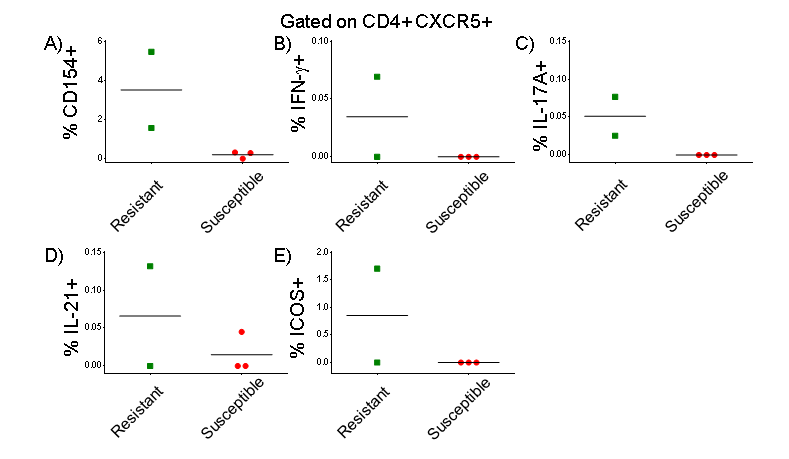

Supplement: S5 Fig — A) Net CD154 expression B-D) Net production of IFN-γ (B), IL-17A (C), IL-21 (D), and net ICOS expression (E) (CFA/I stimulated minus media), D3 –pre-vaccination, by pTfh in Resistant (green squares) and Susceptible (red circles) volunteers. (TIF) [file pntd.0005291.s007.tif]

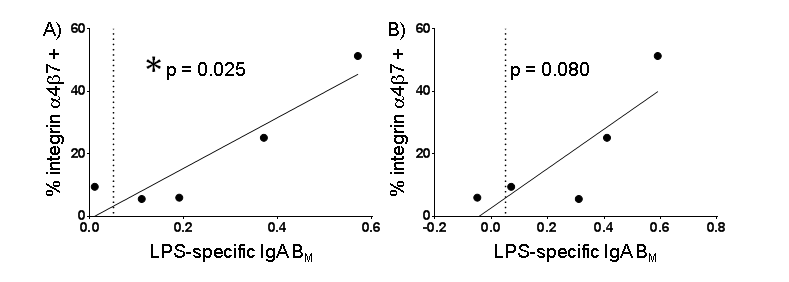

Supplement: S6 Fig — Linear regression analysis comparing percentages of pTfh expressing integrin α4β7 (α4β7+ CCR4-) following stimulation with whole cell homogenate on day 3 post-challenge versus the LPS-specific IgA BM as a percent of total IgA BM on (A) day 14 and (B) day 28 post-challenge. (TIF) [file pntd.0005291.s008.tif]
